# Supplementary material for: The GGDEF protein Dgc2 suppresses both motility and biofilm formation in the filamentous cyanobacterium Leptolyngbya boryana
Source: Microbiol Spectr. 2023 Sep 1;11(5):e04837-22. doi: 10.1128/spectrum.04837-22 (PMC10581220; doi:10.1128/spectrum.04837-22)
Supplement: Supplemental file 1 — Tables S1 to S3, Fig. S1 to S13, and and legends to movies. [file spectrum.04837-22-s0001.pdf]

Supplementary Materials

(Supplementary Tables, Supplementary Figures and Legends to Supplementary Movies)

Name	Sequence	Used
BamHI_Low02920	ggtgatgagatctGGATCCCAGTGTGAGCGTTTTCAG	pIL1007, pIL1018, pIL1019
BamHI_tDGC2_Low	AAAAAGGATCCTTATTCTTCTTCAATCGTTTTCCGTTGTG	pIL1019
Down02920_KmR	GGAATACGATCGGCGaccccagagtcccgc	pIL910
Down02920_pBSAmp	cgaaaagattggGCGCGGAACCCCTATTTG	pIL910
DSR36750_pBR322rop_F	TTTGAATGTAAATCCCCCTTACACGGAGGC	pIL1195
DSR36750_pTrc_R	GAATCTGCACAAACAAttgttatccgctcacaattcc	pIL1195
KmR_and_USR200_F	tagcttgcagtgggcttacatggcg	pIL1195
KmR_Down02920	gggactctggggtCGCCGATCGTATTCCCG	pIL910
KmR_lacI_F	ggttcgaaatgacaccatcgaatggtgc	pIL1195
KmR_Up02920	ccctgcgccatCAAATTTCTTGCCACGGCG	pIL910
KmR_USR35760_R	gcccactgcaagctaTGATTTCCCCGACAATAGAGAC	pIL1195
LacI_KmR_R	tggtgtcatttcgaaccccagagtcc	pIL1195
NSI_DSR_pBRori_F	GCGATCGGACACGGAGGCATCAGTGAC	pIL1205
South_920_L_2	TCCAAGACATCTTGCATCATCACCGTTCG	Southern blotting
South_930_U	ATCTCTGAAAACGCTCACACTGGGAGG	Southern blotting
qPCR_16S_rRNA_F	AGCCAACGCGTTAAGTTGTC	qPCR
qPCR_16S_rRNA_R	AAGGTTCTTCGCGTTGCATC	qPCR
qPCR_00160_F	AACCGTGAAACTGACGCATC	qPCR
qPCR_00160_R	CGTCATCGGGATCGTAACCC	qPCR
qPCR_02920_F_2	ACTTGGAAAACCGCTCGAAG	qPCR
qPCR_02920_R_2	TCATCCTGCTGGTGAAACAC	qPCR
qPCR_LBDG05260_F	TTTGACGTTGCGCTTCGATC	qPCR
qPCR_LBDG05260_R	AGAATTGCAACTGCGTCAGC	qPCR
13120_qPCR_F	TTCGCTGGTGGTAGCATCAG	qPCR
13120_qPCR_R	GCCCATTGCTTCCGAAACTG	qPCR
qPCR_LBDG29990_F	TGGCAACGTCCATTGATCAC	qPCR
qPCR_LBDG29990_R	TGCGATCGCCCGAATTTTAC	qPCR

Table S1. Oligonucleotide primers used in this study.

comparison	t-value	p-value
WT vs. Δ*dgc2*	3.3215	0.04491
WT vs. Δ*dgc2;dgc2ex*	−7.0408	0.005886
WT vs. *dgc2ex*	−1.0479	0.3451

Table S2. Comparison of *dgc2* expression levels.

The expression level of *dgc2* was compared between the wild type and Δ*dgc2,* Δ*dgc2;dgc2ex* or *dgc2ex* strains using qPCR (n = 4). The t-values and p-values of the Welch's t test are shown. A significant difference was found between the wild type and Δ*dgc2* or Δ*dgc2;dgc2ex* strains ($p < 0.05$).

Strain	GenBank Number or NCBI reference Sequence	Identity (%)	E-value	Length (aa)
Leptolyngbya boryana dg5	BAS60749.1			732
Methylacidiphilales sp.	NJL61986.1	48.9	0.0	731
Gracilibacteria sp.	NJK47728.1	33.3	$3e^{-90}$	583
Desulfopila sp. IMCC 35008	WP_163337768.1	29.4	$2e^{-61}$	955
Desulfobulbaceae sp.	HHD63075.1	28.8	$3e^{-61}$	946
Desulfobacterales sp.	PIE64557.1	29.3	$7e^{-61}$	947
Deltaproteobacteria sp.	PIE69581.1	28.7	$5e^{-60}$	687
Competibacteraceae sp.	HAO31409.1	31.2	$1e^{-58}$	951
Contendobacter sp.	KAB2935691.1	32.3	$3e^{-58}$	
Methyloumidiphilus alinensis	PZN72207.1	29.5	$3e^{-56}$	689

Table S3. Bacterial strains harboring genes encoding CHASE2-containing Dgcs.

A BLAST search was performed at NCBI based on the amino acid sequence of Dgc2 (BAS60749.1) in *Leptolyngya*. We selected those with an E-value less than 1e-50 and similar to Dgc2 in their domain component and listed in the table in descending E-value order. The table shows the name of the organism, the GenBank or NCBI reference number, the percentage of amino acid matches at sites similar to Dgc2, the E-value, and the protein length in amino acid (aa) residues.

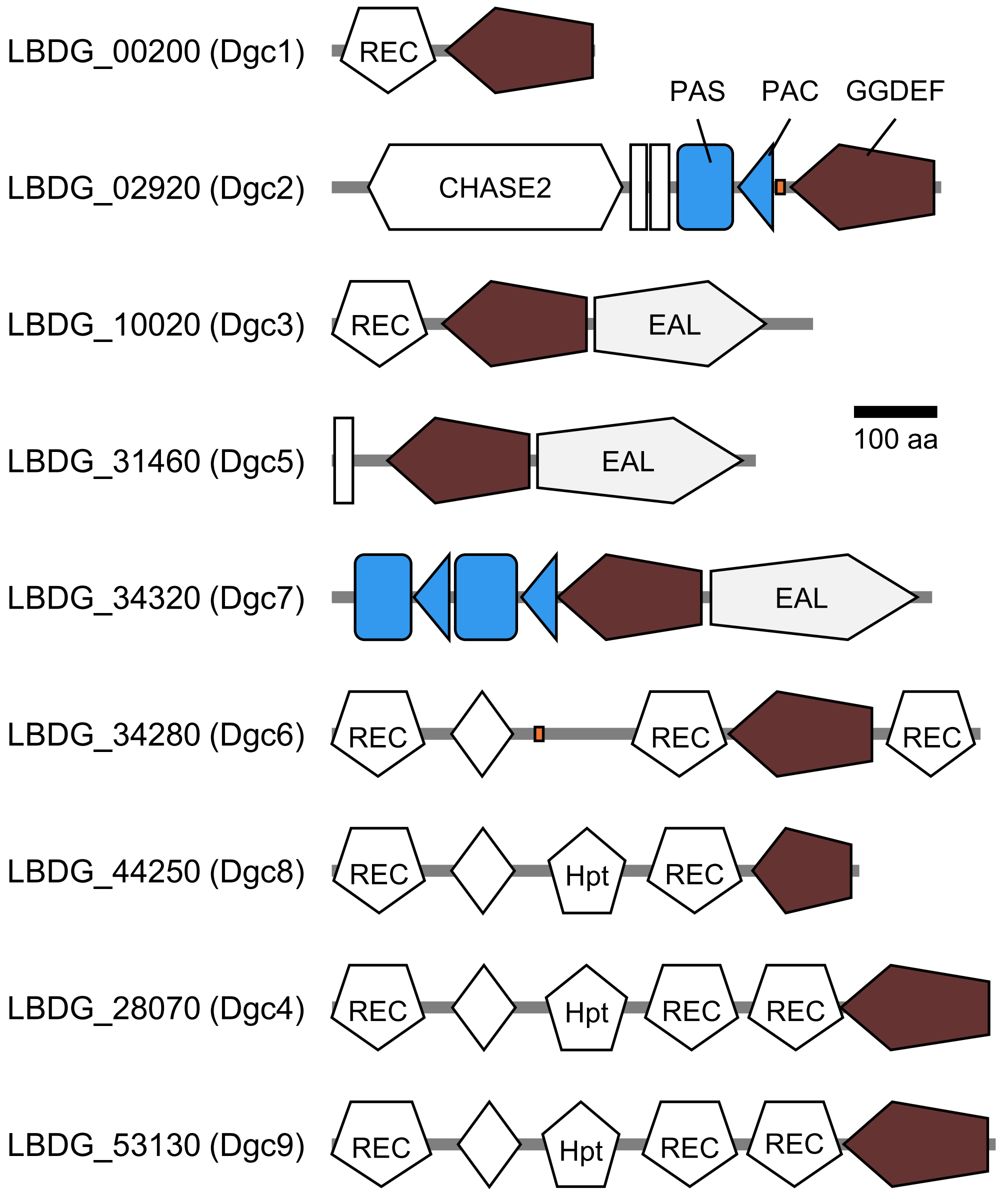

Figure S1. Nine genes found in the *Leptolyngbya* genome encoding proteins harboring the GGDEF motif.

We found nine genes encoding proteins harboring the GGDEF motif. We numbered the ORFs in the order in which they were assigned as "LBDG_" and organized them as Dgc1–9. Dgc1 only has the REC domain, and Dgc3 only has the REC and EAL domains (gray pentagon) in addition to the GGDEF domain. EAL domains were also observed in Dgc5 and Dgc7. Dgc5 harbors the transmembrane region, and Dgc7 contains two PAS and PAC domains. Dgc4, 6, 8, and 9 are similar and contain REC domains and Trans_reg_C (Transcriptional regulatory protein, C terminal; white rhombus). The Hpt domain is also common and found in three genes other than Dgc6.

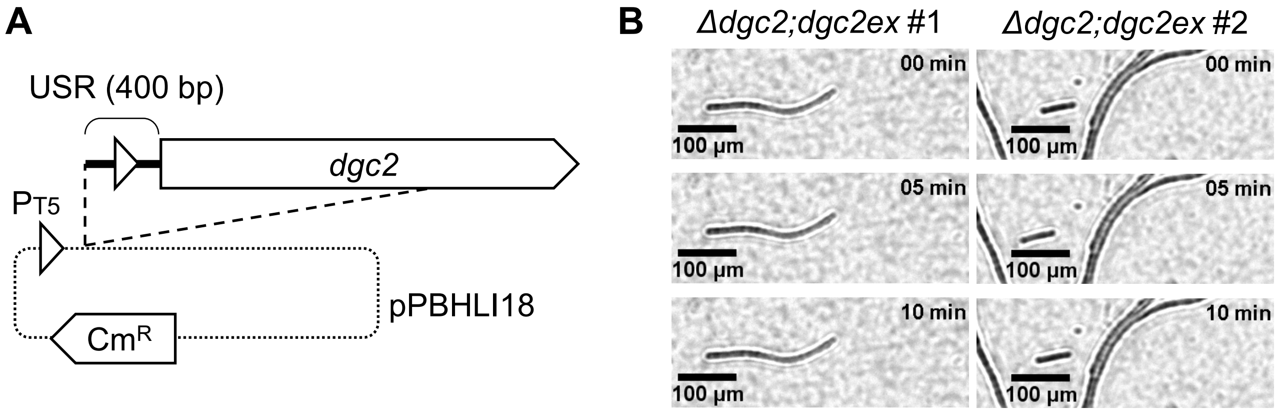

Figure S2. Schematic for ectopic *dgc2* expression and motility of the Δ*dgc2:dgc2ex* strain. A. Schematic representation of the plasmid pIL1007 for ectopic *dgc2* expression. Triangles represent the T5 promoter (P_{T5}) and a possible *dgc2* promoter within the 400-bp upstream region of the gene. Cm^R represents the chloramphenicol-resistance gene. For details, see Materials and Methods. **B**. Most filaments of the Δ*dgc2;dgc2ex* strain were immotile as in the wild type strain (left panels). However, only a single filament showed gliding motility and switching behavior (right panels), see also **Movie S1**, lower two panels.

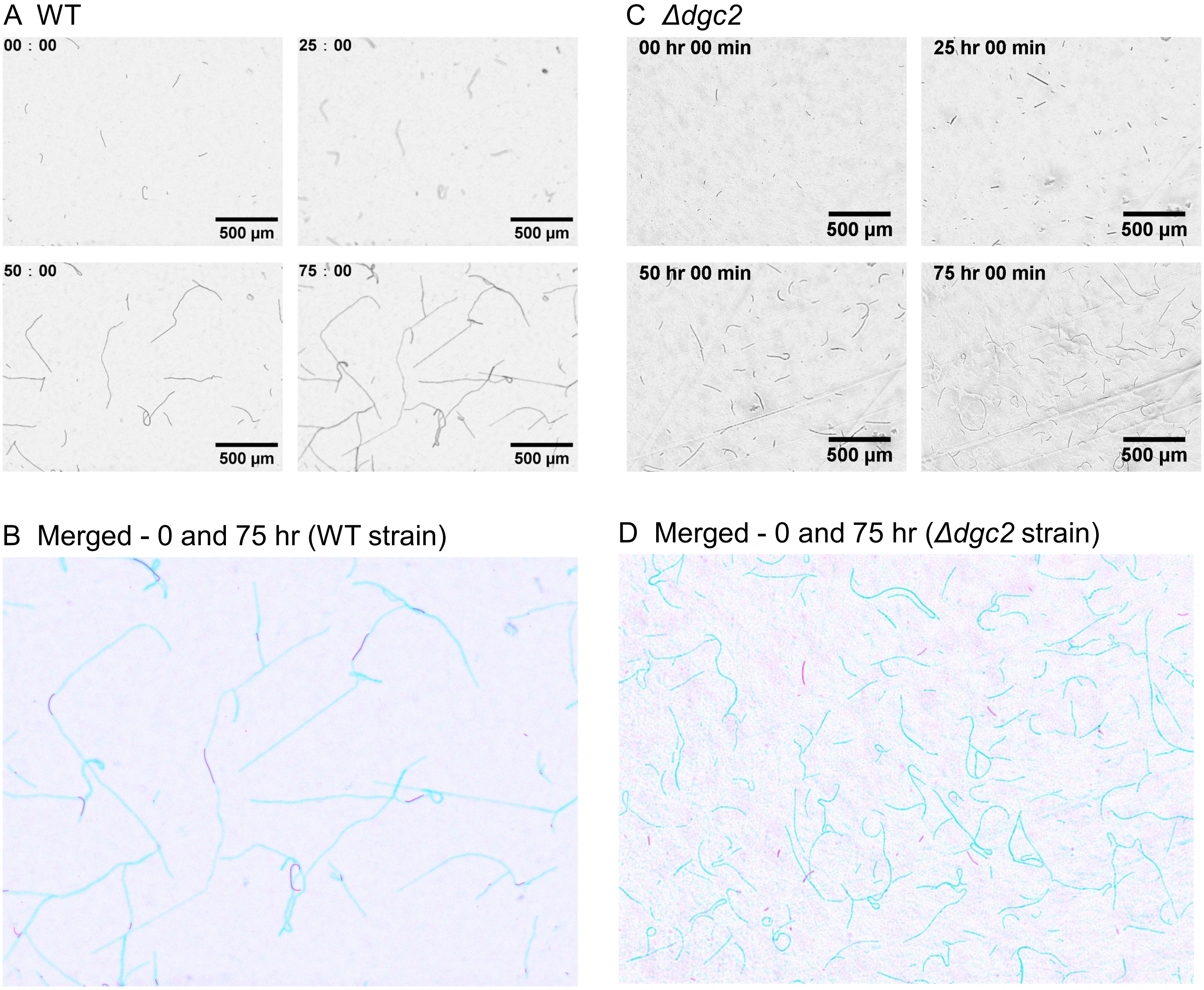

Figure S3. No gliding motility was observed in the wild type strain, but not in the Δ*dgc2* strain. The wild type (**A**) or Δ*dgc2* (**C**) strain was inoculated on agar medium and observed for a long time in the same field of view (**A**). **B** and **D**. Overlaid image of magenta at 0 h and cyan at 75 h. The two overlaid photographs matched well in wild type (**B**), but the ones for Δ*dgc2* did not match at all (**D**). See also **Movie S2**.

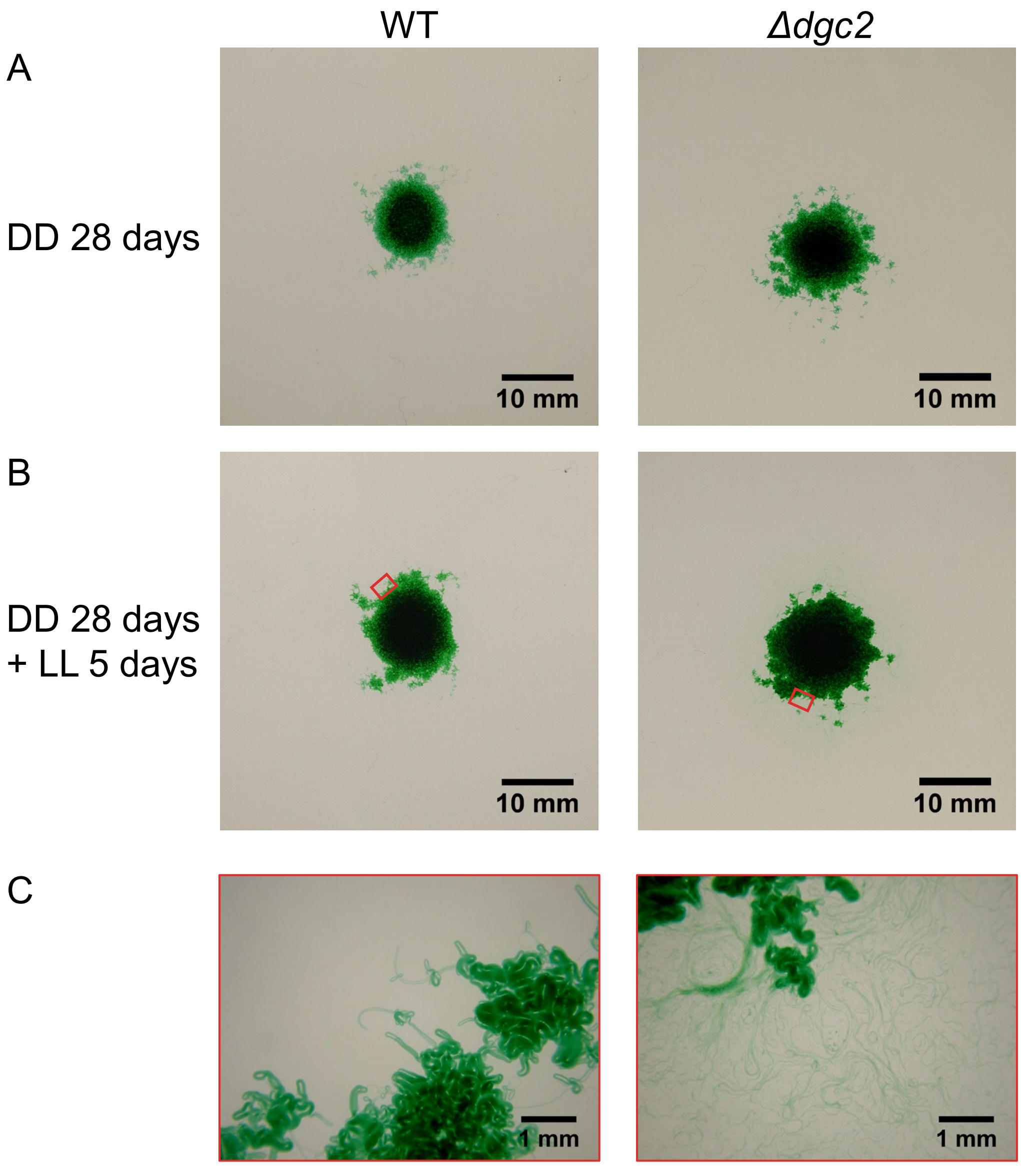

Figure S4. Δ*dgc2* strains also stop gliding motility under heterotrophic conditions. The wild type and Δ*dgc2* strains were grown under heterotrophic conditions for 28 d. Both strains formed ivy-like colonies (**A**). After 5 d under the light, the colony of the wild type strain expanded in a similar shape, whereas the colony of the Δ*dgc2* strain collapsed and expanded to the virgin area (**B**).

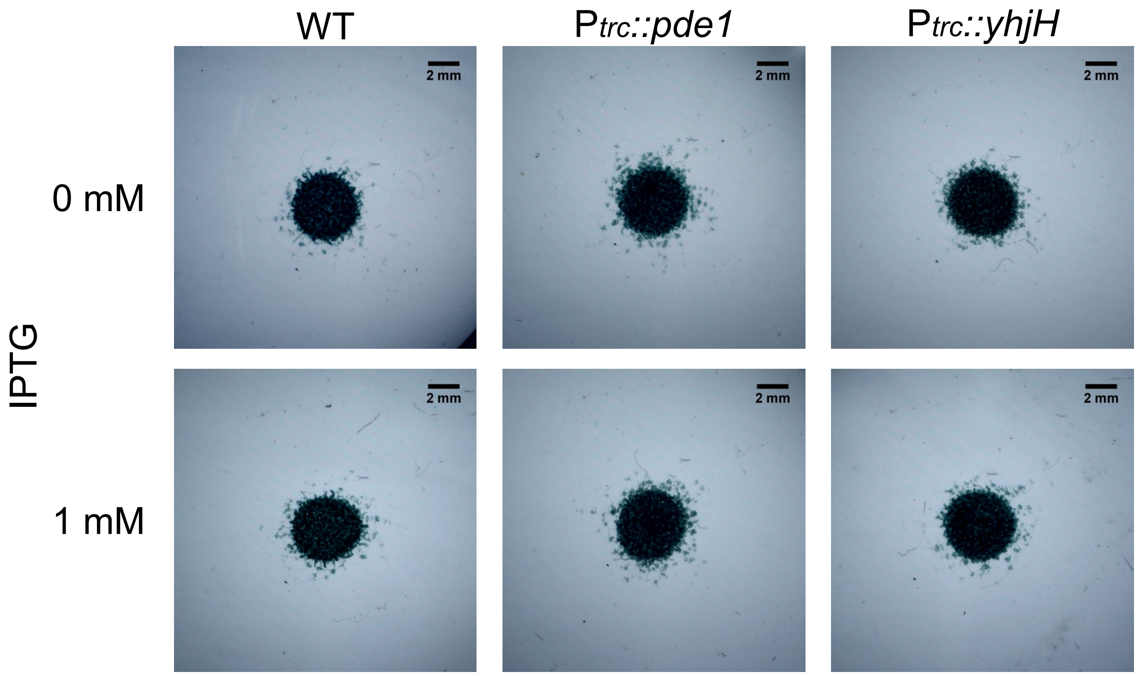

Figure S5. Overexpression of PDEs does not induce motility.
The wild type, P*trc::pde1* and P*trc::yhjH* strains were grown under heterotrophic conditions for 10 d with or without (1 mM) IPTG. All strains formed ivy-like colonies.

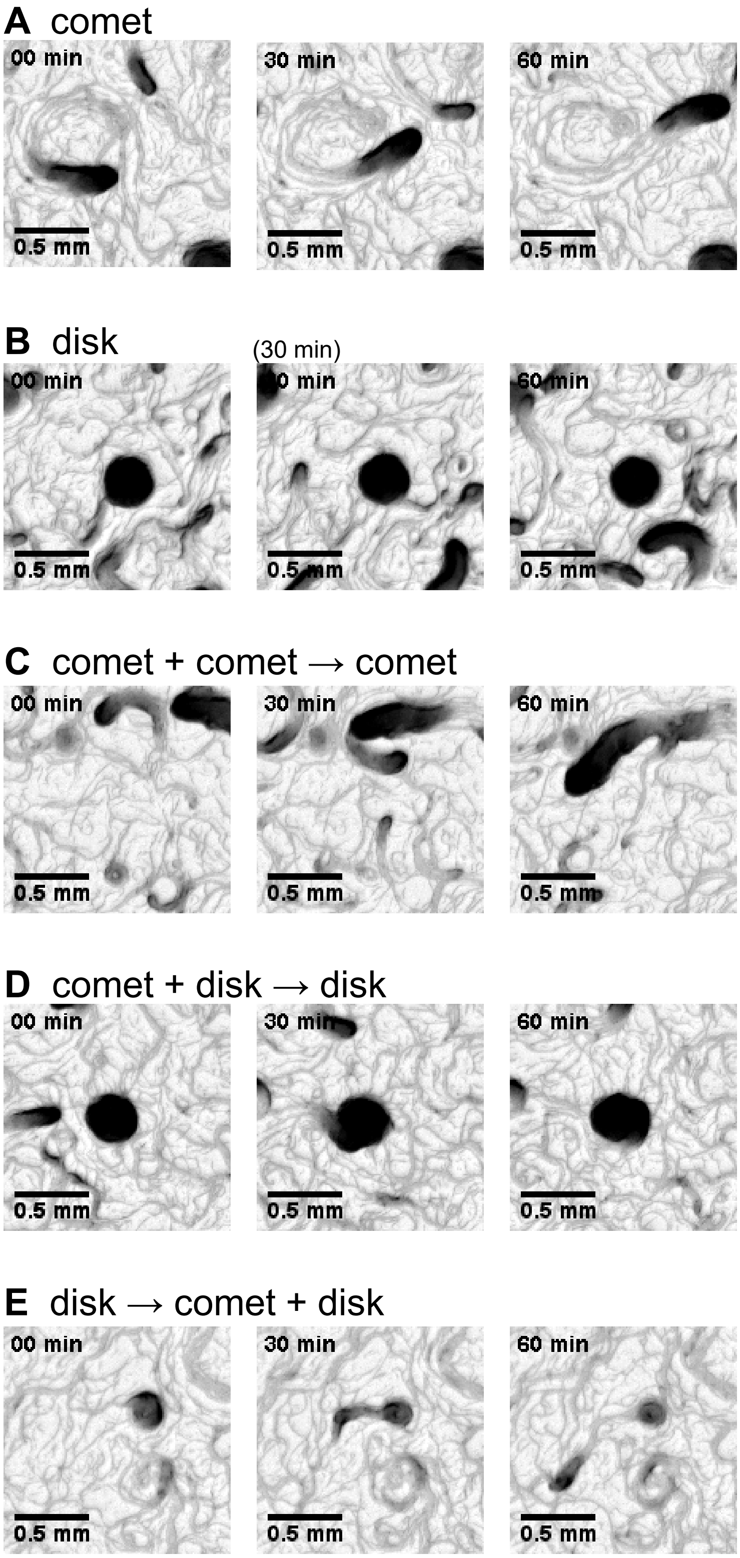

Figure S6. Time-course profiles of the wandering comet-like and rotating disk-like clusters. **A.** Movement and morphology of a comet (extracted from **Movie S4A**). **B.** Movement and morphology of a disk (extracted from **Movie S4B**). **C.** Enlargement of a comet by the unification of multiple comets (extracted from **Movie S5A**). **D.** Enlargement of a disk by integrating with a comet (extracted from **Movie S5B**). **E.** Dissociation of comets from a disk (extracted from **Movie S5C**).

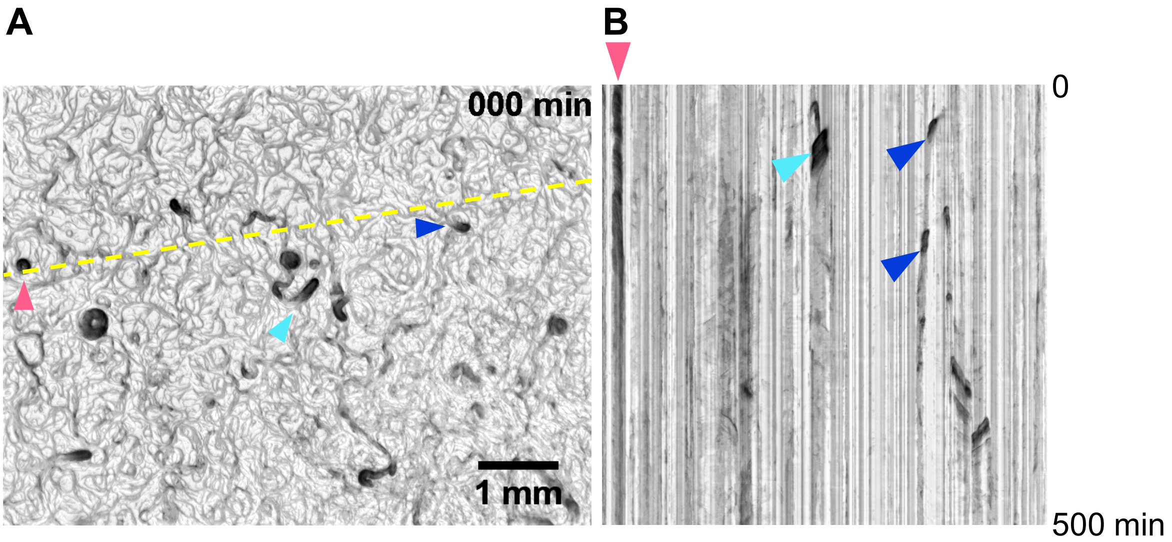

Figure S7. Collective behavior of the original *dgc2*⁻ strain.
A. Time-lapse images of the *dgc2*⁻ strain on solid media. Whole images are summarized in **Movie S3** (left panel). **B**. A kymograph of colonies represented by yellow dashed lines shown in panel **A**. Representation is the same as in **Fig. 6**. Magenta arrowhead represents a rotating disk; cyan and blue arrowheads represent comets.

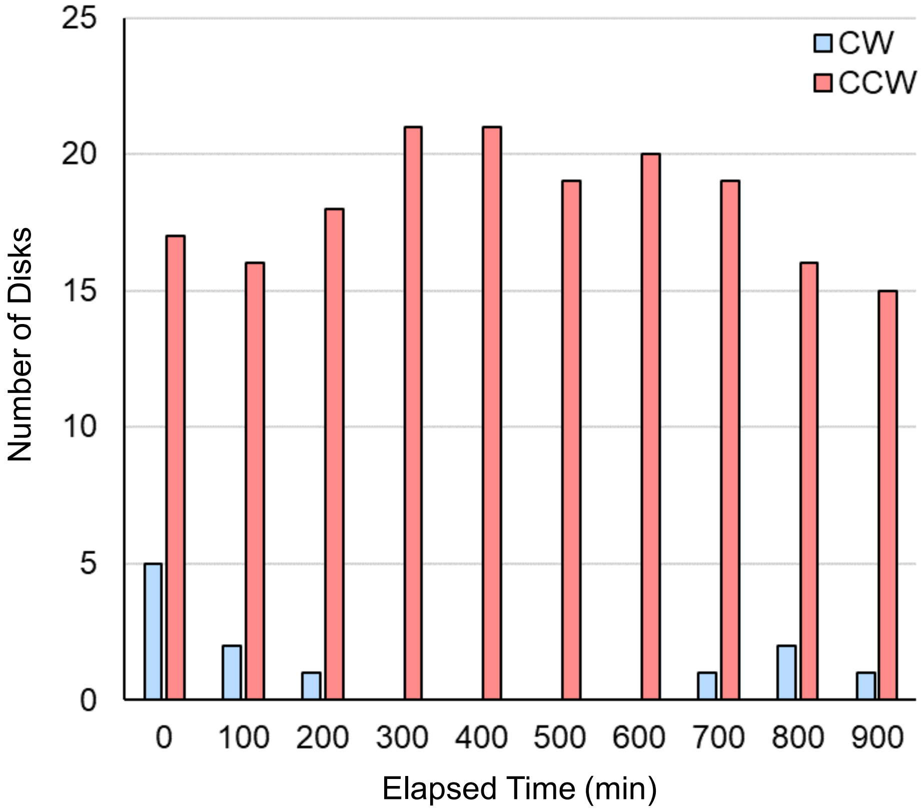

Figure S8. The direction of disk rotation was always CCW-dominant.
The direction of disk rotation was tracked for three frames every 100 min from **Movie S3** (middle and right panel). The blue and red bars indicate the number of disks rotating in the CW and CCW directions, respectively. The disks rotating in the CCW direction were observed more frequently.

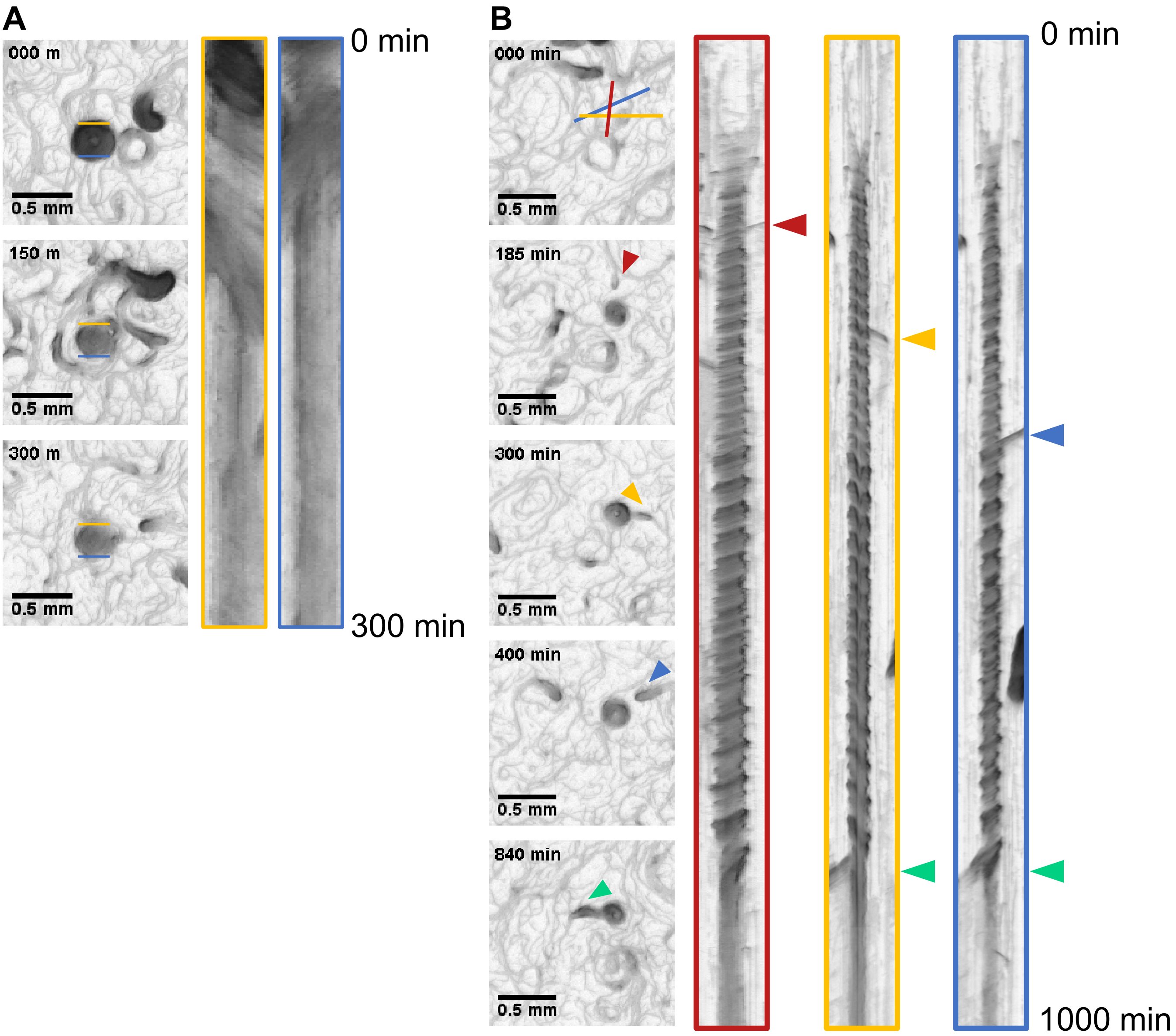

Figure S9. Disintegration of rotating disks.
A. Time-lapse images of a disk rotating in the CW direction in the Δ*dgc2* strain. Kymographs on the upper part (yellow bar) and the lower part (blue bar) of the disk are shown on the left, as represented in **Fig. 6**. The edge of the disk began breaking up into a comet-like cluster. At ~300 min, a smaller number of cells remained at the center, whereas rotation was no longer obvious (see also **Movie S6B**). **B**. Time-course of an exceptional disk cluster rotating in the CCW direction, where the disk disintegrated after collision with some comets. Time-lapse images and three kymographs are shown. The color of the three bars on the image at 0 min are correlated with that of the kymograph. Red and blue arrowheads represent comets which collided with the disk; yellow and green arrowheads represent comets that dissociated from the disk. For details, see text and **Movie S7**.

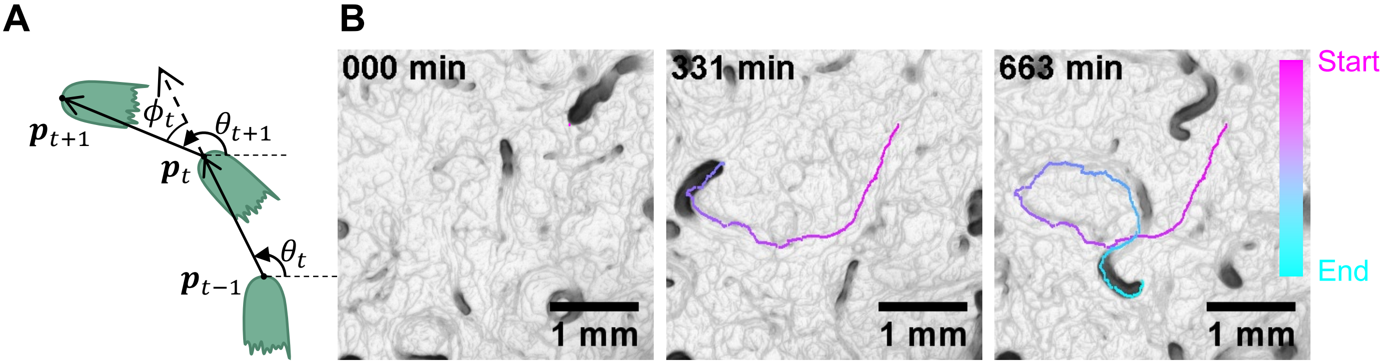

Figure S10. Tracking the trajectory of comets.
A. Schematic diagram of the angular velocity of a comet. p_t, position of the head of a comet at time *t*; $\boldsymbol{v_t} = \overrightarrow{p_{t-1}p_t}$ θ_t; the angle between the velocity vector $\boldsymbol{v_t}$ and the *X*-axis; $\phi_t = \theta_{t+1} - \theta_t$, the angular velocity. **B**. An example of the trajectory of a comet. Colors indicate the positions of the cluster at min 0 (magenta) and 663 (cyan).

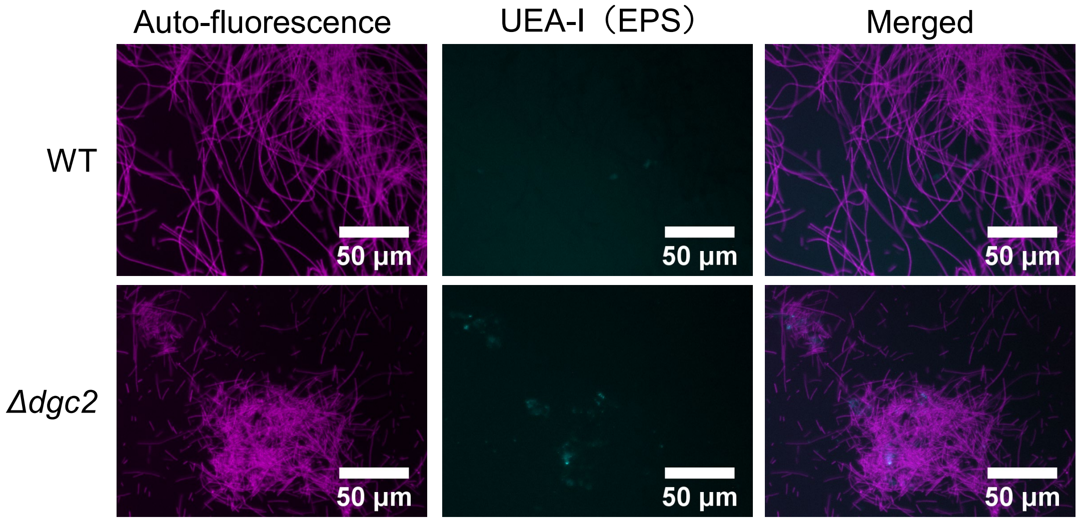

Figure S11. Detection of EPS with UEA-I.
Fluorescence microscopy images of UEA-I-stained Δ*dgc2* cells. Magenta and cyan indicate autofluorescence of cells and stained EPS, respectively.

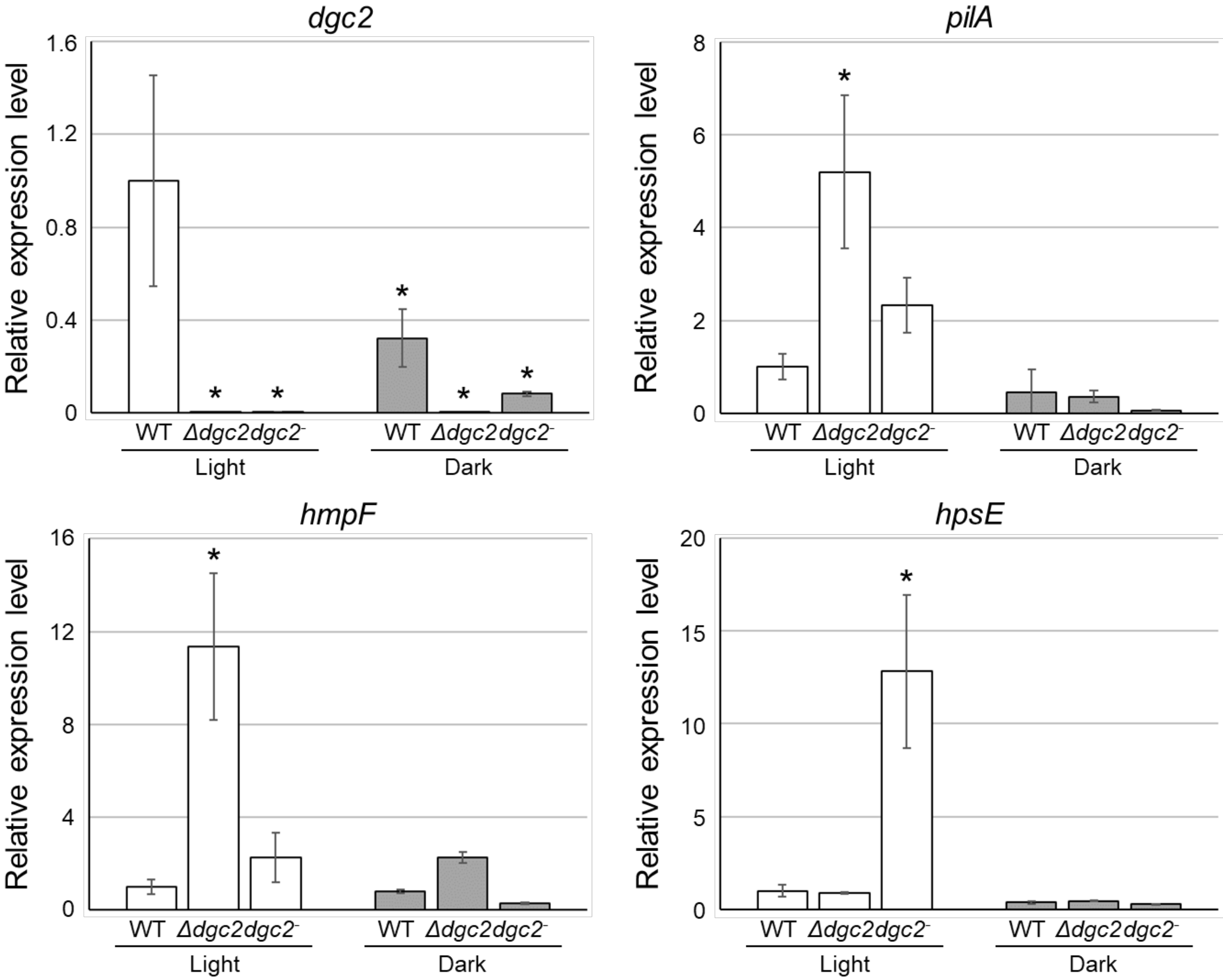

Figure S12. Expression levels of tested genes in the wild type, Δ*dgc2* and *dgc2*⁻strains.
qPCR was performed for four genes, *dgc2*, *pilA*, *hmpF and hpsE* in the wild type, *Δdgc2* and *dgc2*⁻ strains. Expression levels in the wild strain under the light condition were normalized to be 1. Asterisks indicate statistically significant difference in the expression levels between the wild type and each mutant strain by the Dunnett test (p<0.05). For detailed statistical values, see **Table 7**.

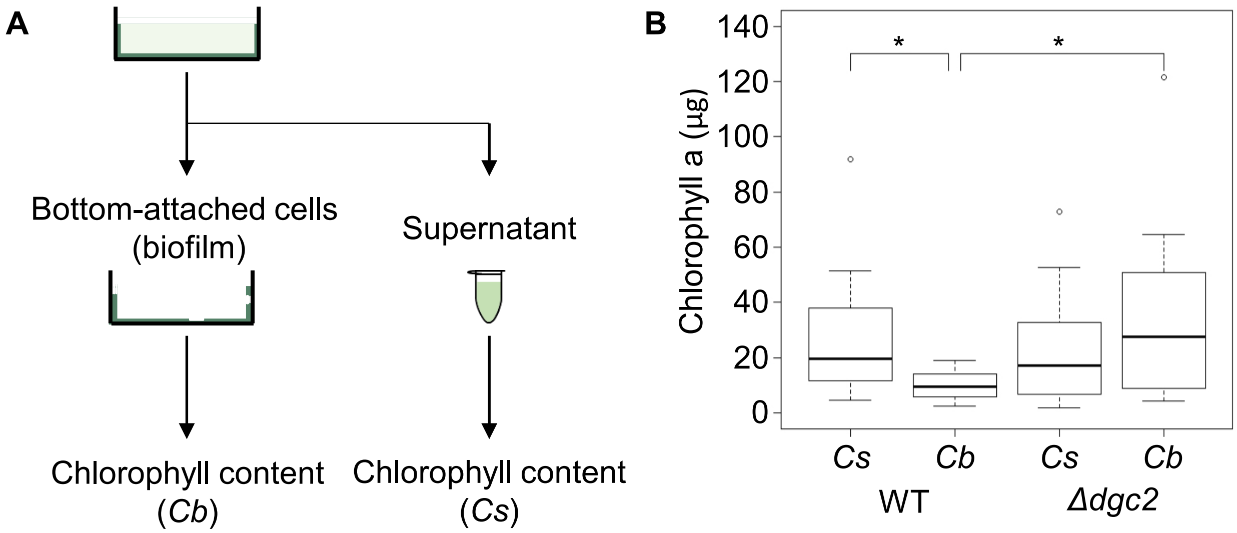

Figure S13. Quantification and distribution of the biofilm.
A. Schematic representation of the experiment to quantify the ratio of bottom-attached cells (biofilm) to the total cell mass (adhesion rate, *Ra*). Cells were cultured in 10 mL of BG-11 medium in a glass-bottom plate. Then, the supernatant was transferred to another tube gently. Chlorophyll content in the supernatant and biofilm were represented as *Cs* and *Cb*, respectively.
B. The amount of chlorophyll extracted from cells attached on the glass-bottom (biofilm; *Cb*) or cells in the liquid phase (*Cs*) in the wild type or Δ*dgc2* strain ($n = 20$ for both strains). Asterisks indicate significant differences between *Cs* and *Cb* from the wild type strain, and between *Cb* in the wild type and in the Δ*dgc2* strain (Welch's *t* test without assuming equal variance, $p < 0.05$).

Legends to Supplementary Movies.

Movie S1. Time-lapse images of motility at the single filament level of the wild type (upper left), *dgc2*⁻ (upper middle), Δ*dgc2* (lower left), and Δ*dgc2;dgc2ex* (lower right) strains on solid media for 20 min.

Movie S2. Observation of the wild type (panel **A**) and Δ*dgc2* (panel **B**) strains for 100 h immediately after inoculation.

Movie S3. Collective behavior of the *dgc2*⁻ (panel **A**) and Δ*dgc2* (panels **B** to **D**) strains on solid media.

Movie S4. Behaviors of a wandering comet-like (panel **A**) and a rotating disk-like (panel **B**) clusters are shown (each extracted from **Movie S3B**).

Movie S5. Transition of migrating cluster types. **A**. Three comets unified into a larger one. **B**. Integration of a comet into a disk. **C**. Separation of a comet from a disk.

Movie S6. Three types of disks showed three different behaviors. **A**. A disk that was hit by comets multiple times but continued to rotate in the CCW direction for 300 min; **B**. A disk that rotated in the CW direction, emitted comet-like clusters, and collapsed; and **C**. A disk that rotated in the CCW direction, but changed direction to CW at 300–350 min, eventually disintegrating to release comet-like clusters.

Movie S7. Rotation of a disk disturbed by collision with comets. The disk collided with other comets at ~185 and 400 min, and comets popped out at ~300 and 840 min.

Movie S8. EPS released from gliding filaments of Δ*dgc2*. India ink was dropped on the filaments of the Δ*dgc2* strain. The trajectory of the filament was stained black with the ink particles.
